# University College, London

**Published:** 1888-10-27

**Authors:** 


### UNIVERSITY COLLEGE, LONDON.

The undermentioned prizes and scholarships have been awarded: Medical Entrance Exhibitions—£100, F. W. Wesley; £60, C. T. Spencer; £40, B. L. Abrahams. Andrew's Entrance Scholarships, £30 each, for—Languages, C. Conroy; Science, S. F. Kirby. Gilchrist Engineering Entrance Scholarship, £35 per annum for two years—P. T. J. Estler. Engineering Matriculation Certificate, S. A. Lang.

